# Progress in Abdominal Surgery

**Published:** 1904-01-09

**Authors:** 


### Progress in Abdominal Surgery.

**The Sensibility of the Peritoneum and Abdominal Organs** has been observed by Lennander [1] during operations carried out wholly or in part under local anæsthesia. The results are tabulated in a manner which will be understood from the following extract: Skin incision, no pain; ligature of vessels in skin, pain; forceps on a vessel and nerve, severe pain; puncture of a distended injected bowel, no pain.

**Cleansing the Peritoneum** by irrigation is preferred by Roberts [2] when many small clots and much fluid are present. For many years the relative values of sponging and irrigating for the purpose of cleansing the peritoneum have been discussed, and even now no unanimous decision has been reached. Experience shows that it is practically impossible to cleanse the peritoneum by irrigation owing to the innumerable pouches which shelter much of the material which it is desired to wash away. Exceptionally, however, there occur cases in which irrigation is capable of efficiently cleansing the cavity. Especially is this so if there is plenty of aseptic fluid with very little adherent exudation, as in cases of ectopic gestation, for then it matters comparatively little that some of the fluid should be left behind in the peritoneal cavity. In cases, however, where there is much adherent exudation on the surface of the bowel irrigation is of very little value, for it fails utterly to remove the adherent flakes. In these cases swabbing must be employed, but the area of the peritoneum is so large that to cleanse thoroughly the whole surface is a very tedious task. Fortunately the peritoneum possesses the power of dealing with a certain amount of microbic infection, and no surgeon will maintain that by any method that we possess is it possible to render aseptic the peritoneum when its whole surface has become infected.

**The Prevention of Ventral Hernia after Laparotomy** depends, according to Wolff,[3] upon whether there has been a greater or less amount of meteorism in the first few days after operation. He argues, therefore, that the mechanical pressure of the distended bowel causes the stitches, especially those in the fascia, to yield, either by the knot giving way or by the thread cutting through the tissue; the skin suture heals by first intention, and remains intact. He therefore only completely closes the wound in cases in which an absolutely certain reactionless result is expected; in other cases he inserts a gauze drain. Hahn replies, and gives a record of 88 laparotomies with suture in three layers without a single ventral hernia, whether meteorism occurred or not. His method is:—(1) Isolated—*i.e.* interrupted—suture of (*a*) peritoneum (plus transversalis fascia); (*b*) aponeuroses of abdominal wall to exclusion of muscles; (*c*) skin with possibly buried sutures of a well-developed panniculus. (2) The sutures should all be of absolutely sterile silk. (3) Complete healing-in of the threads. This means strict asepsis. The skin stitches are removed in about eight days; the patient, as a rule, is up in two weeks, and wears no binder or belt.

**Primary Retroperitoneal Sarcoma,** unconnected in origin with any of the solid viscera of that region are, says Williams,[4] by no means common; and the comparative rarity of records of retroperitoneal sarcoma is illustrated by the fact that in 14,630 tumours collected by Gault, of which 894 were sarcomata, only one case of primary sarcoma of this region is reported. Williams reports an unsuccessful operation on a case. The affection is insidious in its onset, indefinite symptoms of a persistently rebellious and growing interference with the digestive organs are its earliest manifestations. Then come the symptoms due to direct pressure on the nerves and blood-vessels deep in the abdominal cavity. Finally, the rapidly-growing mass reveals itself to the eye and hand. The tumour is usually immovable, is uninfluenced by the action of the diaphragm, and can generally be separated from the liver or the spleen by an area of resonance. The position of the hollow viscera relative to the growth is of great importance in making a physical examination. This is demonstrated by a band of tympany crossing the mass, with dulness on percussion everywhere else over its surface. As the tumour increases in size it separates the leaflets of the mesentery and pushes the intestine forward. Thus, if the tumour is to the right side of the abdomen, the ascending colon is displaced forward and rests on the anterior and left side of the mass. When the growth is centrally located the small intestines are arranged irregularly over the surface of the tumour; the transverse colon rarely crosses the surface of a median tumour, only three cases being recorded. These physical signs and symptoms are not characteristic of sarcoma alone, but may be present with other neoplasms occupying the same area, nor do they exclude the presence of morbid growths originating in the solid organs of the abdomen. The average duration of the condition is seven to eight months from the beginning of the initial symptoms up to the fatal ending of the case. As to treatment, laparotomy should be performed for two reasons:—(1) Exploratory incision is the only sure method of establishing a correct diagnosis; (2) if the possible presence of a tumour is early recognised, a prompt surgical interference may cure the disease or prolong the life of the patient.

[1] Brit. Med. Jour., Nov. 14, 1903, p. 1288. [2] Lancet, Oct. 24, 1903, p. 1180. [3] Glasgow Med. Jour., April, 1903, p. 312. [4] Amer. Jour. Med. Sci., Aug., 1903, p. 269.

